# Temporal and Spatial Differences between Symptomatic and Asymptomatic Malaria Infections in the Chittagong Hill Districts, Bangladesh

**DOI:** 10.4269/ajtmh.21-0121

**Published:** 2022-09-19

**Authors:** Kerry L. Shannon, Timothy Shields, Sabeena Ahmed, Hafizur Rahman, Chai Shwai Prue, Jacob Khyang, Malathi Ram, M. Zahirul Haq, Jasmin Akter, Mohammad Shafiul Alam, Gregory E. Glass, Myaing M. Nyunt, David A. Sack, David J. Sullivan, Wasif A. Khan, Frank C. Curriero

**Affiliations:** ^1^ Department of International Health, Johns Hopkins Bloomberg School of Public Health, Baltimore, Maryland;; ^2^ Department of Emergency Medicine, Johns Hopkins School of Medicine, Baltimore, Maryland;; ^3^ Department of Epidemiology, Johns Hopkins Bloomberg School of Public Health, Baltimore, Maryland;; ^4^ Department of Molecular Microbiology and Immunology, Johns Hopkins Bloomberg School of Public Health, Baltimore, Maryland;; ^5^ Infectious Diseases Division, International Centre for Diarrhoeal Disease Research, Bangladesh, Dhaka, Bangladesh;; ^6^ Laboratory Sciences and Services Division, International Centre for Diarrhoeal Disease Research, Bangladesh, Dhaka, Bangladesh;; ^7^ Health Systems and Population Studies Division, International Centre for Diarrhoeal Disease Research, Bangladesh, Dhaka, Bangladesh;; ^8^ Institute for Global Health University of Maryland Medical School, Baltimore, Maryland

## Abstract

Mapping asymptomatic malaria infections, which contribute to the transmission reservoir, is important for elimination programs. This analysis compared the spatiotemporal patterns of symptomatic and asymptomatic *Plasmodium falciparum* malaria infections in a cohort study of ∼25,000 people living in a rural hypoendemic area of about 179 km^2^ in a small area of the Chittagong Hill Districts of Bangladesh. Asymptomatic infections were identified by active surveillance; symptomatic clinical cases presented for care. Infections were identified by a positive rapid diagnostic test and/or microscopy. Fifty-three subjects with asymptomatic *P. falciparum* infection were compared with 572 subjects with symptomatic *P. falciparum* between mid-October 2009 and mid-October 2012 with regard to seasonality, household location, and extent of spatial clustering. We found increased spatial clustering of symptomatic compared with asymptomatic infections, and the areas of high intensity were only sometimes overlapping. Symptomatic cases had a distinct seasonality, unlike asymptomatic infections, which were detected year-round. In a comparison of 42 symptomatic *Plasmodium vivax* and 777 symptomatic *P. falciparum* cases from mid-October 2009 through mid-March 2015, we found substantial spatial overlap in areas with high infection rates, but the areas with the greatest concentration of infection differed. Detection of both symptomatic *P. falciparum* and symptomatic *P. vivax* infections was greater during the May-to-October high season, although a greater proportion of *P. falciparum* cases occurred during the high season compared with *P. vivax*. These findings reinforce that passive malaria surveillance and treatment of symptomatic cases will not eliminate the asymptomatic reservoirs that occur distinctly in time and space.

## INTRODUCTION

In Bangladesh, malaria is endemic in 13 of 64 districts, with more than 14 million people at risk for infection. The highest rates are in the Chittagong Hill Districts, where *Plasmodium falciparum* is the predominant species and *Plasmodium vivax* occurs to a lesser extent.^1^^,^^2^ Numerous mosquito species transmit malaria in the region.^1^^,^^3^^–^^5^ Symptomatic infections tend to peak from June through August, during the height of the rainy season.^5^^,^^6^ In 2007, the Ministry of Health and the nonprofit group Bangladesh Rural Advancement Committee, as part of the National Malaria Control Program (now the National Malaria Elimination Program), worked to strengthen malaria surveillance and control. They implemented specific interventions such as providing community-based testing and treatment with artemisinin-based drugs and long-lasting insecticide-treated bed nets. A 65% reduction in malaria prevalence and a 91% decrease in malaria mortality was observed between 2008 and 2012, likely as a result of this focused effort.^1^^,^^2^^,^^7^

In 2009, a cohort study—supported by The Johns Hopkins Malaria Institute, in collaboration with International Center for Diarrheal Disease Research, Bangladesh—began in the Chittagong Hill Districts to understand more fully the epidemiology of malaria in the region.^8^ An assessment of symptomatic malaria of this cohort demonstrated a seasonal and spatially clustered epidemic, and identified associated risk factors.^9^ Another assessment demonstrated the particularly high risk of infection among pregnant women with asymptomatic infections occurring year-round.^10^ Further research also found that the vast majority of infections at any given time in the area are sub-clinical rather than clinical.^11^

As more countries shift from highly endemic to hypoendemic settings, finding the remaining hotspots for malaria and responding appropriately is imperative to the success of control and elimination programs. Most of the current strategies are based on locating hotspots of symptomatic, clinically presenting cases. In this article, we explore whether this strategy will likely adequately capture asymptomatic or oligosymptomatic infections that had mild enough symptoms not to present to care by understanding the extent to which symptomatic and asymptomatic infections overlap in space and time. If asymptomatic infections are not occurring in the same geographic pattern or at different times of the year, more active surveillance is then necessary to locate and treat them.

## MATERIALS AND METHODS

**Figure 1.**
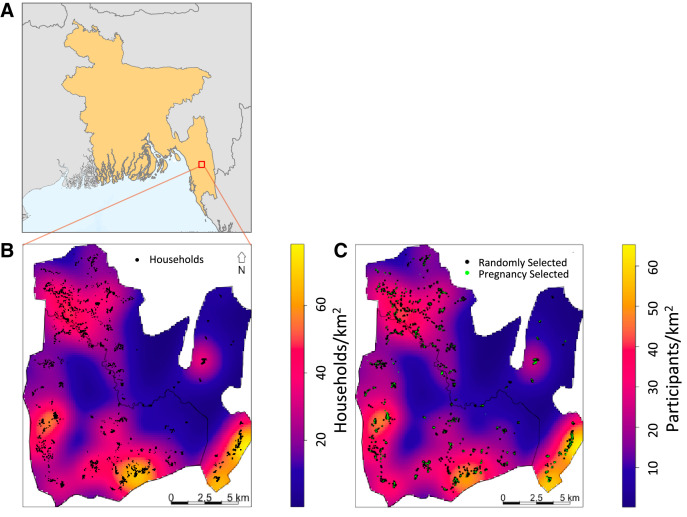
Locator map of study area within Bangladesh (**A**), with the spatial intensity estimates for household concentration in the study area (**B**) and participants in the active surveillance study by selection type (**C**).

This study was conducted in two geographic unions (Kuhalong and Rajbila) located north of Bandarban city in the Bandarban District (Figure 1A). The area is about 179 km^2^ and has approximately 24,000 residents. The methods and structure of the cohort study are explained elsewhere in more detail.^8^ The geographic location and concentration of households per unit area (spatial intensity) in the cohort study was mapped (Figure 1B).

Active surveillance began in mid-October 2009 in Kuhalong Union, and in May 2010 was expanded to include Rajbila Union, and continued through mid-October 2012. Each union was divided into 12 geographic clusters, and one person from each cluster was sampled weekly, rotating between three age groups (0–4, 5–14, and ≥ 15 years). In a year, 624 persons in each union were selected randomly for this active surveillance, or 1,248 each year including both unions. During the 3-year period approximately one sixth of the entire population was actively sampled. Each week, 2 of 12 people were also selected for longitudinal follow-up, with three more visits at 3-month intervals. All pregnant women were also sampled for longitudinal malaria detection during pregnancy. All people sampled provided blood for rapid diagnostic testing and microscopy, and were deemed an asymptomatic infection if positive by either method. All those tested were surveyed for symptoms at time of testing, before results were known. Although we labeled those identified in the passive sample as asymptomatic, a proportion of them were oligosymptomatic, or had mild symptoms that were not enough to result in seeking care. For simplicity, all those testing positive in the active survey are termed asymptomatic infections. For this analysis, we combined all actively sampled persons, including the randomly selected (those selected in the weekly random sampling) and the additional women sampled because they were pregnant. The location of survey participant households was mapped (Figure 1C) and stratified by pregnancy or random selection.

Because of potential biases introduced from oversampling pregnant women, a sensitivity analysis in which all the comparisons between symptomatic and asymptomatic *P. falciparum* infections was conducted for just those randomly selected. This included spatial intensity, seasonality, and clustering analysis.

The passive surveillance system also began in mid-October 2009 in Kuhalong Union, and in May 2010 was expanded to include Rajbila Union and still continues. Infections or cases included were diagnosed with malaria based on a positive rapid diagnostic test (RDT) and/or microscopy when patients sought care for suspected malaria, either through the local Bangladesh Rural Advancement Committee clinic or directly through the study doctor or field workers, which visited each community regularly and were available by cell phone. All cases identified by passive surveillance were symptomatic by nature of presenting with symptoms. For comparison with asymptomatic infections, we limited the follow-up to match the period when active surveillance was conducted (mid-October 2009 to mid-October 2012). For comparing symptomatic *P. falciparum* and *P. vivax* infections, analysis was extended through mid-March 2015.

During visits for both active and passive surveillance, information related to symptoms of malaria was obtained, and a blood sample was collected for a malaria smear for microscopic examination and an RDT (FalciVax; Zephyr Biomedicals, Verna, Goa, India). In a prior study in the Chittagong Hill Tracts of Bangladesh, FalciVax RDT, when compared with real-time polymerase chain reaction (PCR) as the gold standard was found to have a sensitivity of 97.6% and a specificity of 95.8% for *P. falciparum* infections, and a sensitivity of 76.9% and specificity of 100% for *P. vivax* infections. When compared with microscopy as the gold standard, FalciVax had a sensitivity of 98.2% and a specificity of 97% for *P. falciparum* infections, and a sensitivity of 90.5% and a specificity of 99.7% for *P. vivax* infections.^12^

Household locations were captured using a global positioning satellite device. The latitude and longitude coordinates were converted to Universal Transverse Mercator zone 46N for analysis using ARCGIS 10.1 (Environmental Systems Research Institute, Redlands, CA).^13^

The main aspects of the analysis focused on the spatial intensity, spatial clustering, and potential effects of seasonality 1) comparing asymptomatic and symptomatic *P. falciparum* infections, and 2) comparing symptomatic *P. falciparum* and *P. vivax* infections.

### Comparison of the spatial intensity, clustering, and seasonality of symptomatic and asymptomatic *Plasmodium falciparum* infections.

Spatial intensity (the expected number of infections per square kilometer) was estimated for symptomatic and asymptomatic malaria infections using a non-parametric kernel density approach and was mapped to highlight spatial variation in the concentration of infections by household location.^14^^,^^15^ The ratio of these respective spatial intensities was mapped to characterize the spatial variation (spatial odds) in expected numbers of asymptomatic infections relative to symptomatic cases. The output compares the intensity of prevalent asymptomatic infections found by active surveillance (a subset of what was present in the entire population) to the total incident symptomatic cases in the population captured by passive surveillance. Because not all asymptomatic infections in the population were sampled, the comparison of the locations of higher to lower spatial intensities as captured in the relative value of the odds ratio rather than the absolute value of the odds is most useful. This intensity analysis was performed for all infections and stratified by season (high season, May–October; low season, November–April).^8^

Spatial clustering describes how dispersed or tightly compact a set of mapped events are, complementary to spatial intensity. It characterizes spatial variation in the location and concentration of events. The K-function was used to estimate the expected number of infections within various distances of other infections of the same type (i.e., symptomatic or asymptomatic) to assess spatial clustering.^14^ The difference in K-functions (K asymptomatic – K symptomatic) examined the extent to which their spatial clustering differed over a range of distances. The K-function clustering analysis was performed for all *P. falciparum* infections and was then stratified by season. Significant differences in spatial clustering were assessed using the Monte Carlo random-labeling approach.^16^

The cross K-function is similar to the K-function, but it assesses the spatial interaction between two sets of event locations by estimating for a range of distances the expected number of one type of event around the second type. The cross K-function assessed the extent of clustering of asymptomatic infections around symptomatic cases. The results were compared under the null hypothesis that asymptomatic and symptomatic infections are spatially independent.^17^ Because the asymptomatic infections come from a sampled subset of the population and not the entire population of the cohort (as is true for the symptomatic cases), the number expected at a given distance from a symptomatic case was extrapolated to the entire population. For this analysis, the number of asymptomatic infections was multiplied by the inverse of the proportion of the population sampled to estimate the total number of asymptomatic infections likely present in the entire population at a given distance from a symptomatic case.

### Comparison of the spatial intensity, clustering, and seasonality of symptomatic *Plasmodium falciparum* and *Plasmodium vivax* infections.

Using spatial intensity and K-function methodology, the spatial intensity and clustering of symptomatic *P. falciparum* and *P. vivax* infections captured through the passive surveillance system from October 2009 through March 2015 were compared. Because both were incident cases, the spatial odds was used to estimate and visualize the spatial variation in risk of each type of infection. Seasonality was assessed by comparing proportions of infection during the high and low seasons, and a Z-test compared proportions.

Data were analyzed using R 3.1.1 (R Core Team, Vienna, Austria) and ArcGIS 10.1.^13^^,^^18^ R packages included spatstat, maptools, splancs, maps, SDMTools, and GISTools.^19^^–^^24^

## RESULTS

Of the 3,978 people surveyed by active screening, 3,971 (99.8%) had laboratory information available. Demographics of this group and the cohort study population are shown in Table 1. Of these participants, 54 asymptomatic infections were identified with rapid diagnostic testing and/or microscopy. Of these, 53 (98.1%) were positive *P. falciparum* infections and one (1.9%) was positive for *P. vivax* infection by microscopy with no co-infections. Thirty-five of the asymptomatic *P. falciparum* infections were identified by random sampling and 18 were detected during pregnancy surveillance. The geometric mean parasite density of the 44 microscopy-positive infections was 902 parasites/μL (range, 40–14,600 parasites/μL). Of those participants sampled randomly as positive for asymptomatic *P. falciparum* malaria infection, 68.6% were noted to have fever, headache, fatigue, and/or muscle aches in the prior 2 weeks compared with 17.5% of those who tested negative (odds ratio [OR], 10.3; 95% CI, 4.8–23.4). Of those who were pregnant, 85.0% of those who tested positive for *P. falciparum* malaria infection had fever, headache, fatigue, and/or muscle aches in the prior 2 weeks compared with 48.8% in those who tested negative (OR, 5.9; 95% CI, 1.7–31.9).

**Table 1:** Demographics of cohort study population and those selected into active surveillance study

Household factors	Cohort study, *n *(%)	Asymptomatic study, *n *(%)
Union		
Rajbila	11,139 (44.4)	1,807 (45.4)
Kuhalong	13,955 (55.6)	2,171 (54.6)
Gender		
Male	12,210 (48.7)	1,616 (40.6)
Female	12,884 (51.3)	2,362 (59.4)
Age		
0–59 months	3,170 (12.6)	863 (21.7)
5–14 years	5,785 (23.1)	1,282 (32.2)
15–39 years	10,764 (42.9)	1,346 (33.8)
≥ 40 years	5,375 (21.4)	487 (12.2)
Ethnicity		
Bengali	5,090 (20.3)	821 (20.6)
Total tribal	20,004 (79.7)	3,157 (79.4)
Marma	15,159 (60.4)	2,367 (59.5)
Tanchangya	2,229 (8.9)	357 (9.0)
Khyang	1,204 (4.8)	209 (5.3)
Chakma	879 (1.7)	133 (3.3)
Tripura	431 (1.7)	64 (1.6)
Other tribal	102 (0.4)	27 (0.7)
Education level*		
0–2 years	9,295 (57.6)	1,051 (57.3)
3–5 years	2,999 (18.6)	355 (19.4)
≥ 6 years	3,845 (23.8)	427 (23.3)
Total	25,094	3,978

* Age = 15+ years; *n* = 16,139 for the cohort study and *n* = 1,833 for the asymptomatic study.

In the passive survey between mid-October 2009 and mid-March 2015, there were 812 people with documented malaria by RDT and/or microscopy, 770 (94.8%) with *P. falciparum* mono infections, 35 (4.3%) with *P. vivax* mono infections, and seven (0.9%) with *P. falciparum *and* P. vivax* co-infections. Of the 812 positive cases, 808 (99.5%) were positive by RDT and 655 (80.7%) were positive by microscopy, with 95 (11.7%) not having complete blood films and one (0.1%) not having a documented RDT.

Among passively detected symptomatic *P. falciparum* cases, 572 (73.6%) were documented during the same period the asymptomatic infections were detected by active survey (mid-October 2009 to mid-October 2012) and were used as the comparison group for assessing differences between symptomatic and asymptomatic infections. A total of 570 (99.7%) were positive by RDT, whereas 456 (79.7%) were positive by microscopy, and 63 (13.8%) did not have completed blood films. The geometric mean parasite density of the 456 symptomatic microscopy-positive infections was 4,740 parasites/μL (range, 100–144,000 parasites/μL).

Household location data were available for 52 of the 53 asymptomatic *P. falciparum* infections. For the passive survey, spatial information was available for 39 of the 42 *P. vivax* infections and 763 of the 777 *P. falciparum* infections (565 of the 572 from mid-October 2009 to mid-October 2012). Infections without spatial information were dropped from the mapping and spatial intensity aspects of this analysis but were kept for seasonality statistics.

### Comparison of the spatial intensity, clustering, and seasonality of symptomatic and asymptomatic *Plasmodium falciparum* infections.

**Figure 2.**
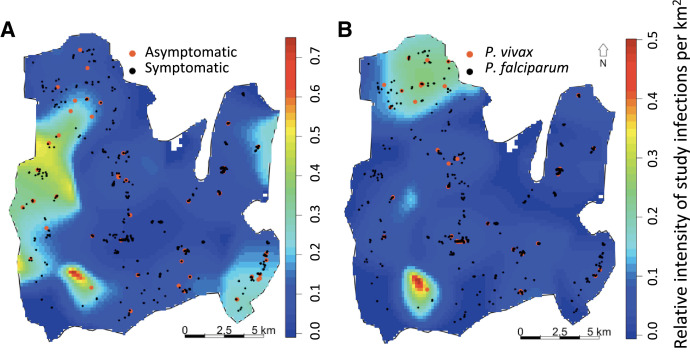
The spatial odds for asymptomatic to symptomatic Plasmodium falciparum infections. A comparison of the intensity of symptomatic cases relative to the intensity of detected asymptomatic infections (**A**) and the spatial odds for symptomatic Plasmodium vivax to Plasmodium falciparum infections (**B**).

The spatial odds of asymptomatic *P. falciparum* infections to symptomatic cases was structured, with areas in darker blue in Figure 2A representing regions with more symptomatic cases and few asymptomatic infections. The central blue area represents a large symptomatic hotspot. Areas in yellow and red represent those with a higher ratio of asymptomatic infections compared with symptomatic cases.

**Figure 3.**
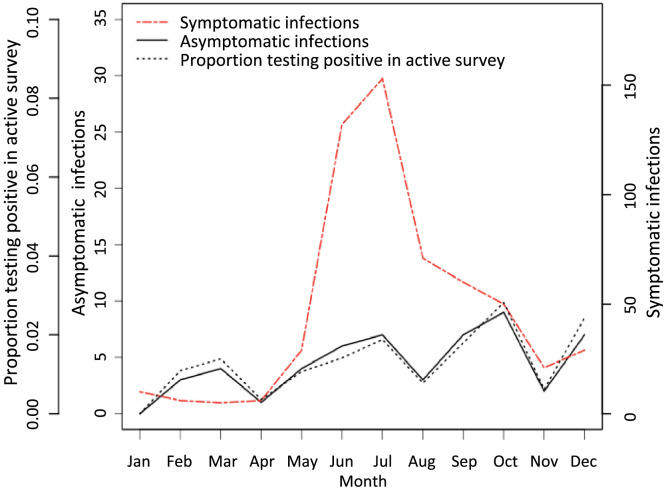
Symptomatic and asymptomatic *Plasmodium falciparum* infections detected by month through the active and passive surveillance systems, and the proportion testing positive in the active surveillance survey.

There was pronounced seasonality in the symptomatic cases when compared with asymptomatic infections. Symptomatic cases had an extensive peak in June and July, whereas the asymptomatic infections were detected year-round (Figure 3). For passive-case detection, of the 572 positive symptomatic cases, 495 (86.5%) occurred during the May to October high season and 77 (13.5%) occurred during the November to April low season. Similar percentages were found when analyzing the more extensive data through March 2015. For the active surveillance, 36 of the positive asymptomatic infections (68%) were detected during the high season, making up 1.6% of the 2,200 people tested during this period, and 17 (32%) were detected during the low season, accounting for 1.0% of the 1,771 people surveyed during this season.

**Figure 4.**
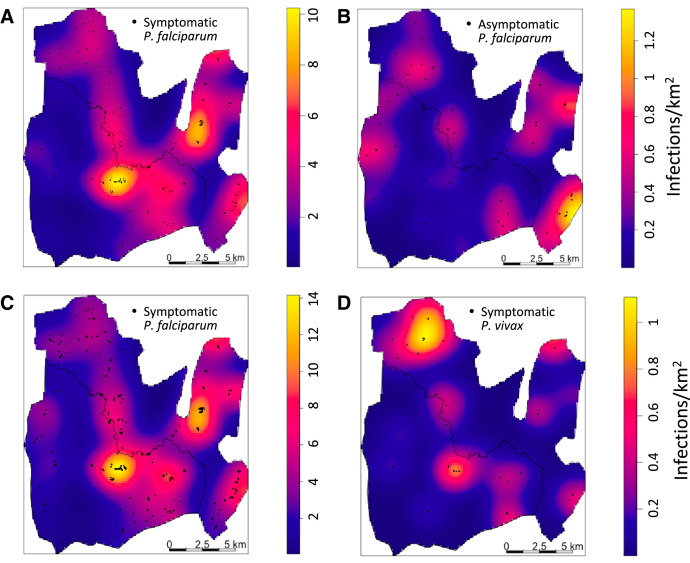
Spatial intensities of symptomatic and asymptomatic *Plasmodium falciparum* infections detected by season from October 2009 to 2012 (**A**,** B**) and spatial intensities of symptomatic *Plasmodium falciparum* and *Plasmodium vivax* infections from mid-October 2009 to March 2015 (**C**,** D**).

The regions of greater risk intensity differed between asymptomatic and symptomatic infections (Figure 4A and 4B). Although generally lower case numbers occurred in the low season, areas of greater symptomatic case intensity were similar across seasons, but differed from the areas of high asymptomatic intensities (Supplemental Figure 1).

**Figure 5.**
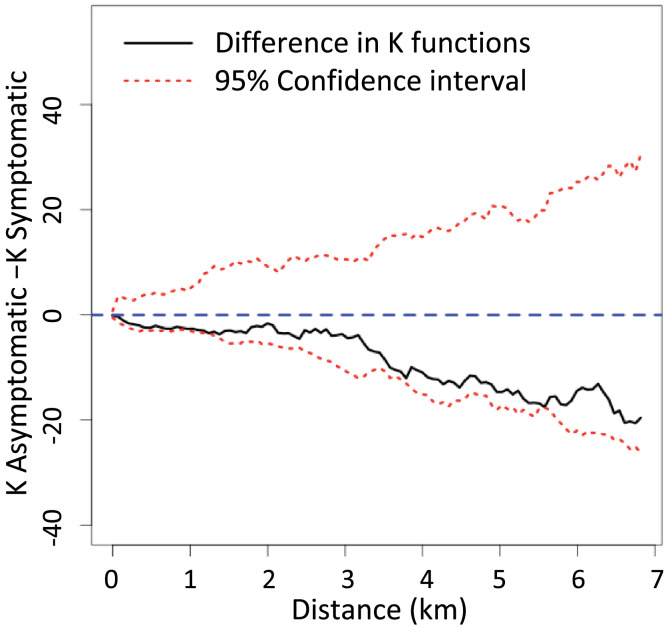
Clustering of asymptomatic and symptomatic infections by difference in K-functions.

The patterns between symptomatic and asymptomatic infections presented in the maps (Figure 4A and 4B) are suggestive of increased symptomatic case clustering (more spatially compact cases) compared with asymptomatic infections, as measured by the differences in the K-functions, both overall (Figure 5) and seasonally (Supplemental Figure 2) at distances up to between 1 and 2 km. These differences were more evident during the high season (Supplemental Figure 2A) than during the low season (Supplemental Figure 2B).

**Figure 6.**
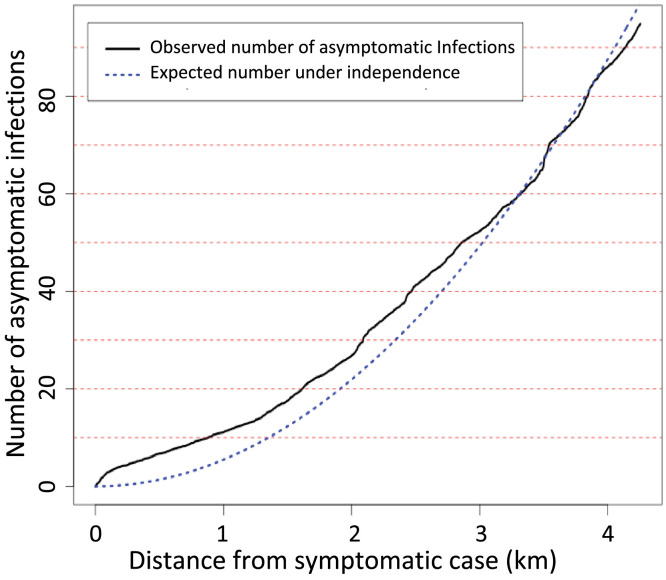
Cross K-function showing the average number of asymptomatic infections in the population within varying distances from a symptomatic case.

The cross K-function (Figure 6) demonstrates the extent of clustering of an actively detected asymptomatic infection around any symptomatic case. There may have been a slight increase in the number of asymptomatic infections near symptomatic cases up to about 2.5 to 3 km. However, the increase was small, with an average of only four to five more infections than would be expected if they were spatially independent, suggesting only a very small increase in asymptomatic infections within 2.5 to 3 km of symptomatic case locations.

The sensitivity analysis (excluding samples selected due to pregnancy) showed few differences from the primary analysis. There was one area of greater intensity of pregnant woman with asymptomatic malaria infections in the southeastern region of the map that, when excluded, favored high intensity of symptomatic cases (Supplemental Figure 3). The difference in K-functions in the sensitivity analysis showed greater spatial clustering at borderline significance out to about 2.5 to 4 km compared with 1.5 km seen in the primary analysis year-round and during the high season, and borderline significance during the low season at around 5 to 6 km not seen in the primary analysis (Supplemental Figure S4). However, the overall conclusions, as earlier, were unchanged.

### Comparison of the spatial intensity, clustering, and seasonality of symptomatic *Plasmodium falciparum* and *Plasmodium vivax* infections

Comparisons for *P. falciparum* and *P. vivax* were only conducted for symptomatic clinical cases because of the detection of only one asymptomatic *P. vivax* infection in the active survey. For this comparison, cases found between October 2009 and March 2015 were used. As noted earlier, 94.8% of the symptomatic cases were *P. falciparum *infections, 4.3% were *P. vivax*, and 0.9% were co-infections. Thus, overall, the spatial odds of *P. vivax* infection relative to *P. falciparum* symptomatic infection indicate a greater likelihood of *P. falciparum* infection than a *P. vivax* infection (Figure 2B). However in a few areas the ratio was more than 0.2, suggesting that there was a substantial contribution of *P. vivax* to malaria infections in these areas. The spatial intensities of clinical symptomatic *P. falciparum* and *P. vivax* cases (Figure 4C and 4D), as well as seasonal changes (Supplemental Figure S5), indicate a substantial overlap in the areas of greater intensity of infection, although the areas with the greatest rates differed, with the northwestern corner of the map in northern Rajbila Union having the greatest intensity of *P. vivax* infection whereas the center of the map in northern central Kuhalong Union had the greatest intensity of *P. falciparum* infection.

The difference in K-function comparing symptomatic *P. falciparum* and *P. vivax* (Supplemental Figure S6) showed no significant difference in *P. vivax* compared with *P. falciparum* cases overall and during the high season, and mostly no difference but borderline increased clustering of *P. vivax* compared with *P. falciparum* cases during the low season up to 1 km.

A greater proportion of symptomatic *P. falciparum* compared with symptomatic *P. vivax* infections occurred during the high season (*P* = 0.0002, two-proportion Z-test with continuity correction). However, both increased during the high season, with 661 *P. falciparum* cases (85%) compared with 26 *P. vivax* cases (62%) occurring during this time.

## DISCUSSION

This study using only rapid diagnostic testing and microscopy demonstrated that asymptomatic infections may not occur at the same time and place as symptomatic infections, differing both in seasonality as well as geographic locations. This possibility is absolutely critical to consider when designing interventions. In prior analyses, we noted that the majority of infections in the area were asymptomatic when comparing passive to active incident data.^11^ Consequently, although there is some overlap in geography, only using symptomatic malaria to target control strategies would fail to identify regions where asymptomatic infections occur—and these regions may subsequently re-seed intervention sites. As such, a strategy for active surveillance is necessary for elimination.

The study also saw a general decrease in the number of symptomatic infections seen in the later years of the study compared with earlier years. This may very well relate to the large push of national control programs in the area.

The factors that contribute to an infection being symptomatic rather than asymptomatic are complex. Environmental, behavioral, and genetic factors may influence exposure, immunity, and access to clinical care for the populations at risk. However, we suspect, in our study, the presumptive ease of access and availability of the study clinician to make house calls meant that these barriers likely did not decrease substantially the number of people who sought care, and the development of symptoms was likely a more important predictor of a clinical disease. It is possible there are different isolates of malaria parasites that have varying degrees of virulence, possibly impacting the development of symptoms. In our study, there was an increase in geometric mean parasite density among the symptomatic compared with asymptomatic infections. More research is needed to characterize the role of virulence, with a focus on those strains that do have the potential to cause clinical disease.

With respect to *P. falciparum* and *P. vivax* infections, there were some areas of overlap in the hotspots. However, the areas with the greatest number of *P. vivax* infections were not the most common areas for *P. falciparum*. These differences may be based on the preferred habitats of vector species that carry these infections, although more research is necessary.^3^^,^^25^

There are several limitations to this analysis. First, the lack of PCR means that a number of sub-microscopic infections could have been missed in the active survey. However, the sensitivity of the FalciVax RDT was estimated at 97.6% for *P. falciparum* and 76.9% for *P. vivax* compared with real-time PCR. We added microscopy secondarily to improve sensitivity. We thus are likely to catch the large majority of *P. falciparum* infections, but may be missing a slightly greater fraction of *P. vivax* infections.^12^ The use of RDT makes the study more practical for real-time implementation in countries with limited resources.

Second, in comparing just the asymptomatic infections and symptomatic cases, we assumed that the sampling in the active study reflects the population in general. Their recruitment does appear similar to the distribution of the general population (Figure 1). Although pregnant women were oversampled, removal of this subpopulation during the sensitivity analysis indicated relatively little impact on overall patterns. The small numbers of asymptomatic infections also made it challenging to define spatial relationships among subgroups or across seasons. A larger study including, in particular, a larger group of asymptomatic infections could be helpful for more detailed modeling.

Another limitation relates to the nature of the active survey data collection. Only one person a week was surveyed in each of the 24 geographic clusters for our prevalence survey. As a result, clusters in the same space and time may have been missed. This issue is mitigated in part by the long period of infection likely for most of those in the active survey.^11^

Furthermore, the asymptomatic infections, rather than the proportion testing positive, were compared during this analysis. The infection intensity is reasonably reflective of the intensity of the proportion that was positive, because the population was sampled randomly in the active survey and all pregnant women, regardless of location, were included. Nevertheless, it is possible the sampling design introduced some bias.

For both passive and active surveillance, the coverage from October 2009 to April 2010 was for only one (Kuhalong) of the two unions. Thus, the number of infections picked up was less than during the same period in following years and may have been slightly weighted spatially to Kuhalong. However, this would impact both passive and active studies equally, and impact detection of *P. falciparum/P. vivax* infections equally, so spatial odds ratios should not be affected.

In conclusion, the majority of *P. falciparum* infections in the area were found to be asymptomatic and persisted year-round. Although the symptomatic and asymptomatic hotspots overlapped, symptomatic hotspots did not predict consistently areas of ongoing asymptomatic transmission, particularly during the low season. It is thus necessary to develop active surveillance for elimination programs.

## Supplemental files


Supplemental materials
