# Supplementary material for: Temporal and Spatial Differences between Symptomatic and Asymptomatic Malaria Infections in the Chittagong Hill Districts, Bangladesh
Source: Am J Trop Med Hyg. 2022 Sep 19;107(6):1210–7. doi: 10.4269/ajtmh.21-0121 (PMC9768271; doi:10.4269/ajtmh.21-0121)

Supplemental Figure 1: Intensity of symptomatic (a-b) and asymptomatic (c-d) *P. falciparum* infections detected by season from October 2009-2012

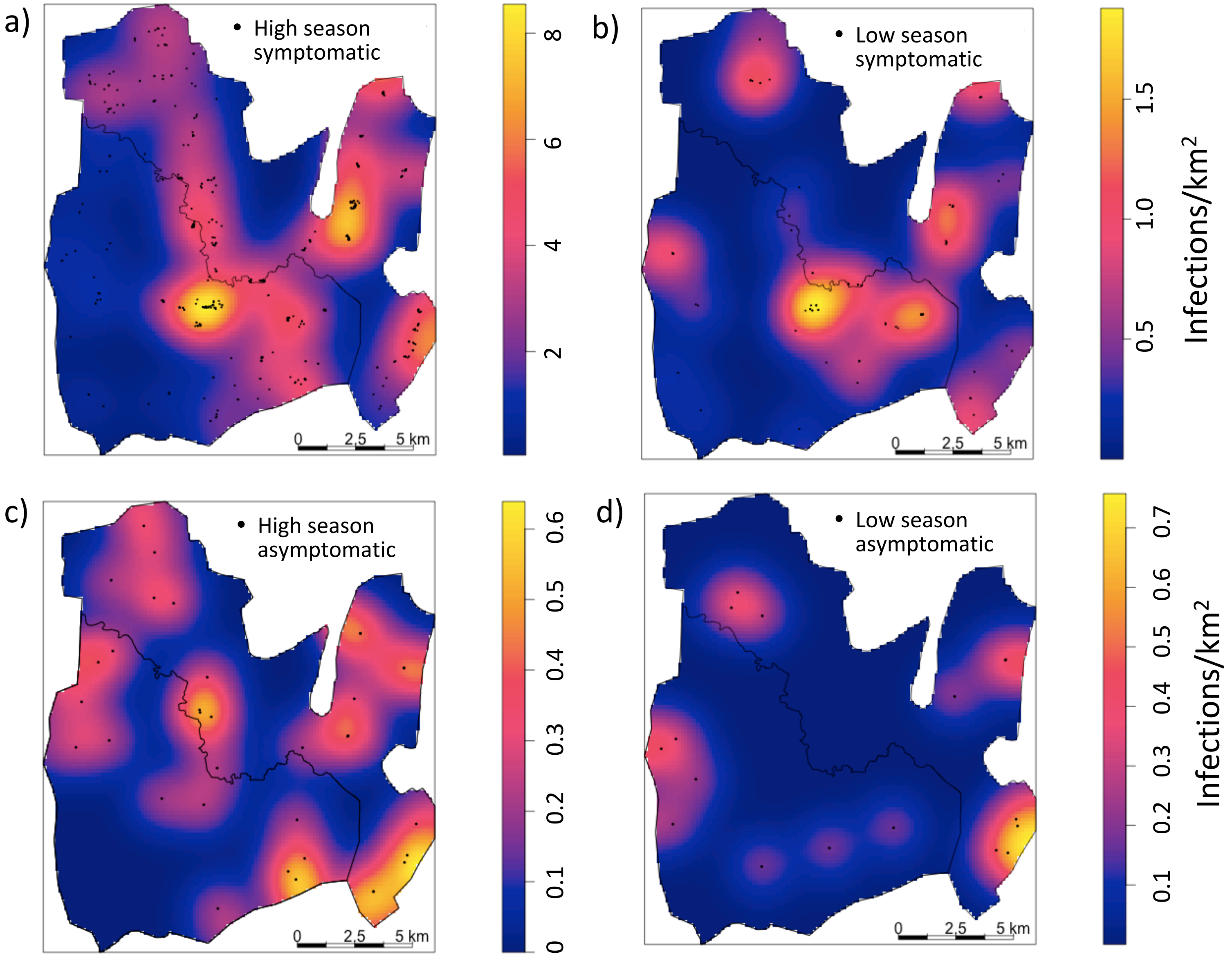

Supplemental Figure 2: Clustering of asymptomatic and symptomatic infections by difference in K-functions during high season (a) and low season (b)

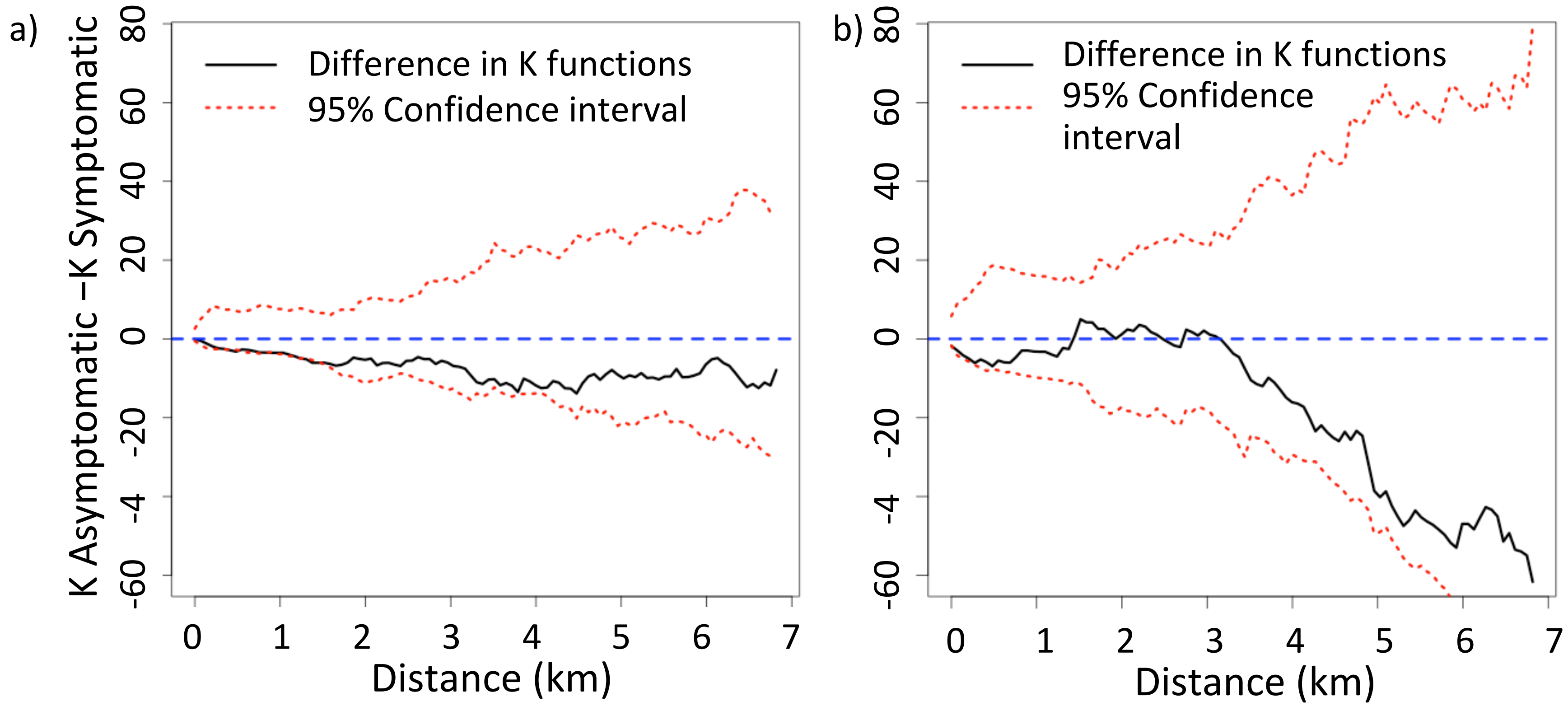

Supplemental Figure 3: Sensitivity analysis, estimated spatial odds of asymptomatic relative to symptomatic infections excluding pregnancy sampled population

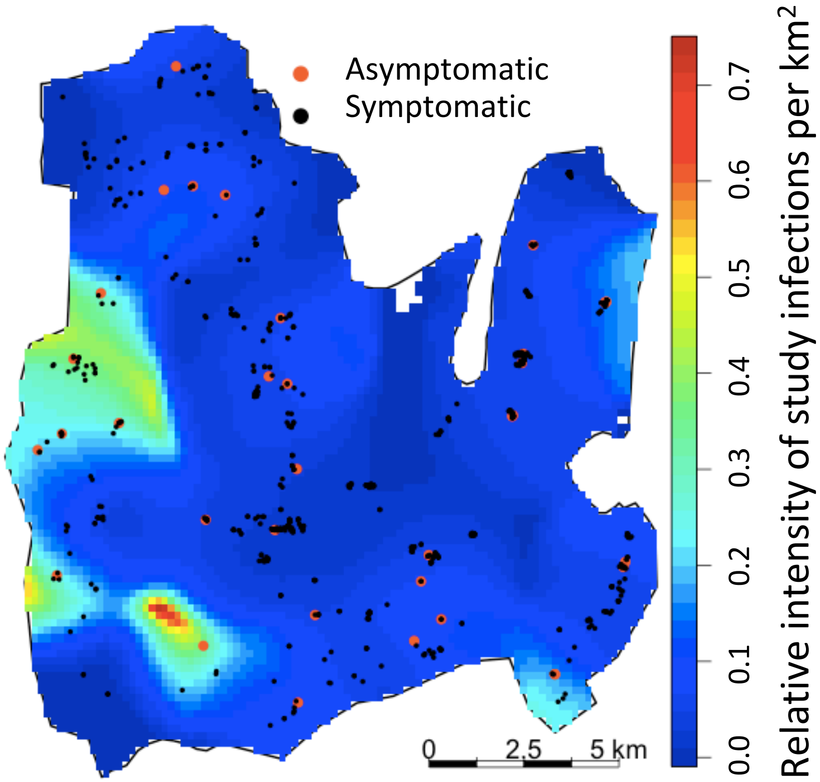

Supplemental Figure 4: Sensitivity analysis, clustering of asymptomatic and symptomatic infections by difference in K-functions overall (a) and during high season(b) and low season(c)

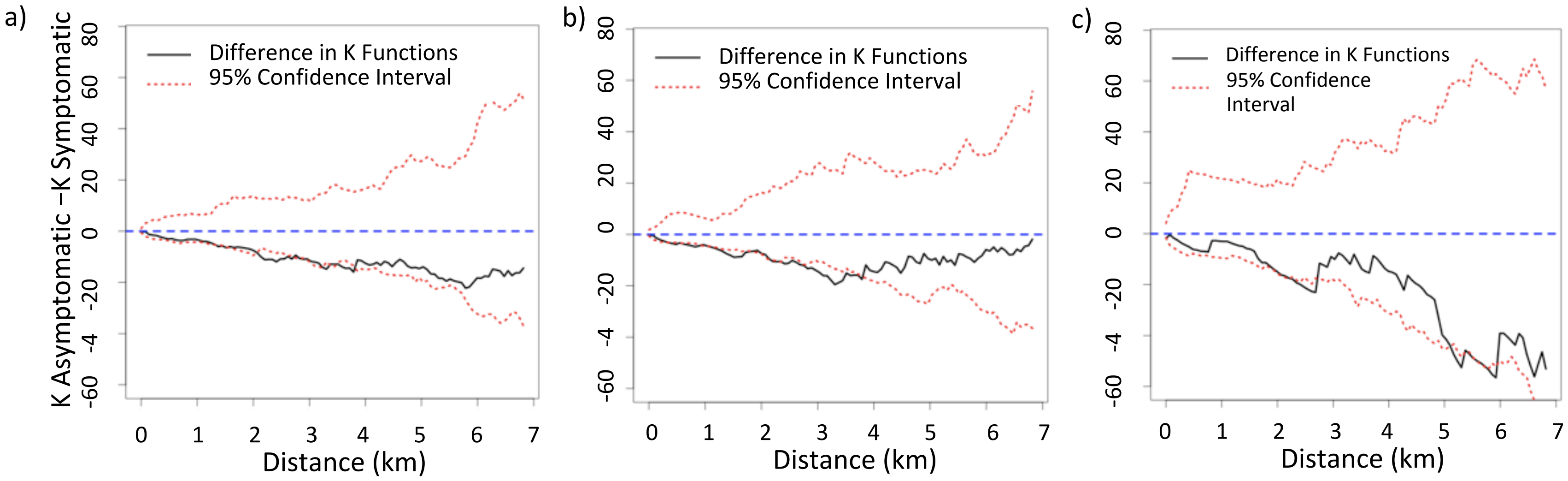

Supplemental Figure 5: The spatial intensities of symptomatic *P. falciparum* (a-b) and *P. vivax* (c-d) infections by season from mid-October 2009-March 2015

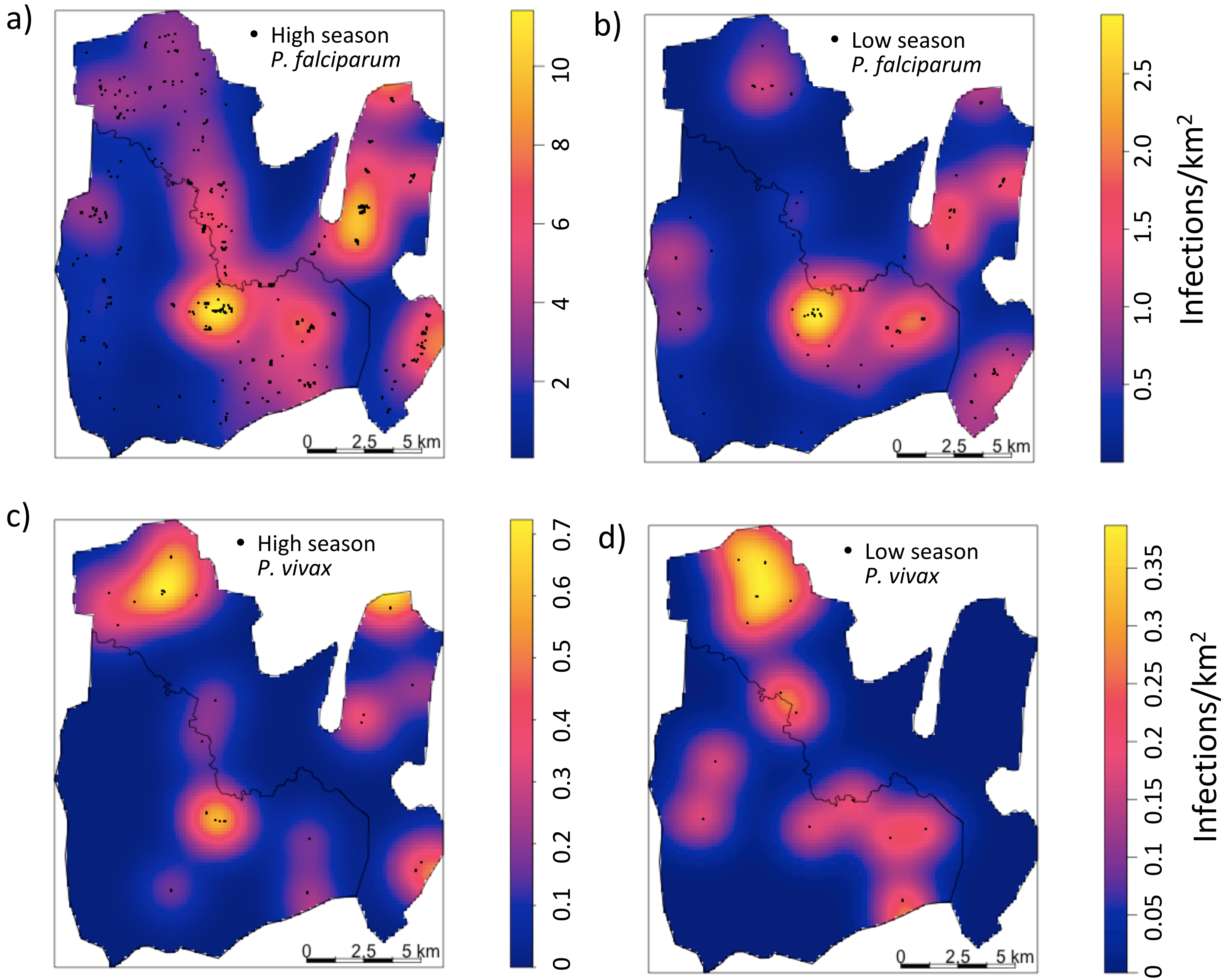

Supplemental Figure 6: Clustering of *P. vivax* and *P. falciparum* cases by difference in K-functions overall(a) and during high season(b) and low season(c)

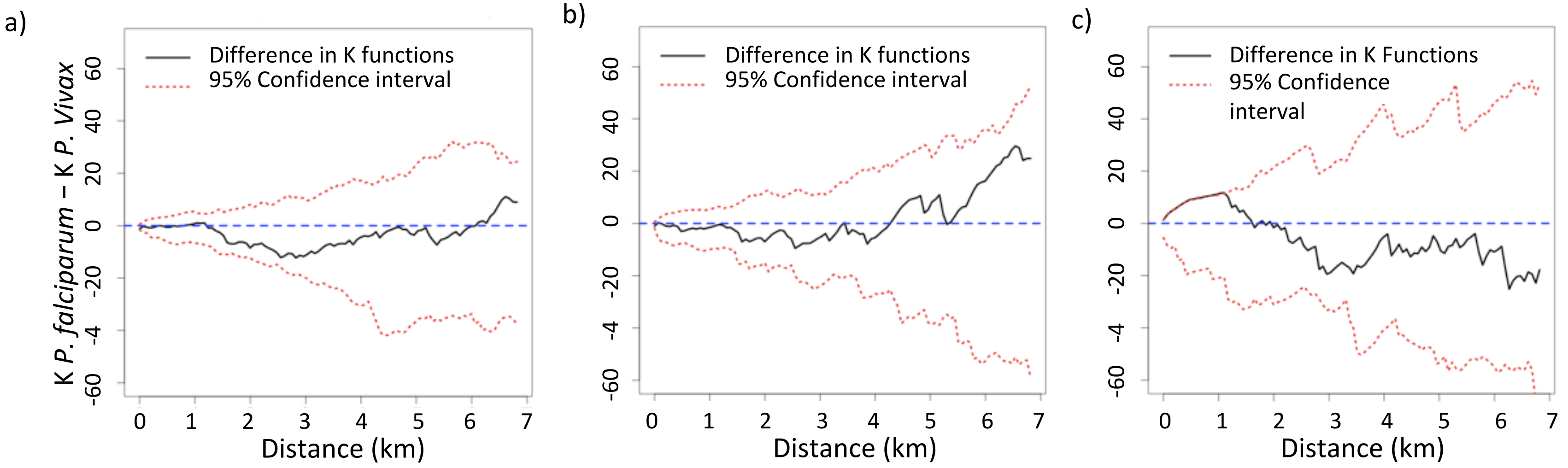

Supplement: Supplementary file 1 [file tpmd210121.SD1.pdf]
